# Heat waves may trigger unexpected surge in aerosol and ozone precursor emissions from sedges in urban landscapes

**DOI:** 10.1073/pnas.2412817121

**Published:** 2024-10-21

**Authors:** Hui Wang, Sanjeevi Nagalingam, Allison M. Welch, Christopher Leong, C. I. Czimczik, Alex B. Guenther

**Affiliations:** ^a^Department of Earth System Science, University of California, Irvine, CA 92697

**Keywords:** isoprene, heat wave, ozone

## Abstract

Biogenic isoprene emissions from herbaceous plants are generally lower than those from trees. However, our study finds widespread isoprene emission in herbaceous sedge plants, with a stronger temperature response surpassing current tree-derived models. We measured and compared isoprene emissions from sedges grown in different climatic zones, all showing an exponential temperature response with a Q10 range of 7.2 to 12, significantly higher than the Q10 of about 3 for other common isoprene emitters. The distinct temperature sensitivity of sedges makes them a hidden isoprene source, significant during heat waves but not easily detected in mild weather. For instance, isoprene emissions from *Carex praegracilis* can increase by 320% with a peak emission of over 100 nmol m^−2^ s^−1^ compared to preheat wave emissions. During heat waves, the peak isoprene emissions from *C. praegracilis* can match those from *Lophostemon confertus*, a commonly used street tree species which is considered the dominant urban isoprene source due to higher biomass and emission capacities. This surge in isoprene from globally distributed sedges, including those in urban landscapes, could contribute to peak ozone and aerosol pollutants during heat waves.

Isoprene is a reactive volatile organic compound (VOC), emitted at high rates from terrestrial ecosystems, that plays an important role for air quality and climate (1). Isoprene is an important precursor of ozone and aerosol in the troposphere, and it can also affect the oxidation capacity of atmosphere and influence the lifetime of methane, a potent greenhouse gas (2–5). Plants synthesize isoprene from dimethylallyl diphosphate (DMADP) with isoprene synthase (IspS) through the methyl erythritol 4-phosphate (MEP) pathway, a process mainly controlled by light and temperature (6). Quantifying the temperature control of isoprene emission from plants has been a fundamental research effort since plant isoprene emissions were first identified (7). Based on emission measurements of a wide range of tree species, an average isoprene Q10 value of about 3 is currently used in atmospheric chemistry models. Q10 represents the change in isoprene emissions with a 10 °C change in leaf temperature (6). However, recent studies reported a high-temperature sensitivity of isoprene from boreal and arctic ecosystems with a Q10 over 8, and it was shown that sedges (i.e., *Eriopohorum* spp. and *Carex* spp.) are the cause of that high-temperature sensitivity (8). Considering the extreme environment of the Arctic, it would be not surprising if arctic sedges displayed a different behavior compared to other isoprene emitters. However, we observed the same distinct temperature sensitivity in other sedge species common to temperate climates in this study, which demonstrates that the high-temperature sensitivity is widely distributed in sedges, at least *Carex* spp. The results of this study suggest a previously overlooked source of isoprene, which has significant implications for air quality and earth system modeling.

## Results and Discussion

**Fig. 1.**
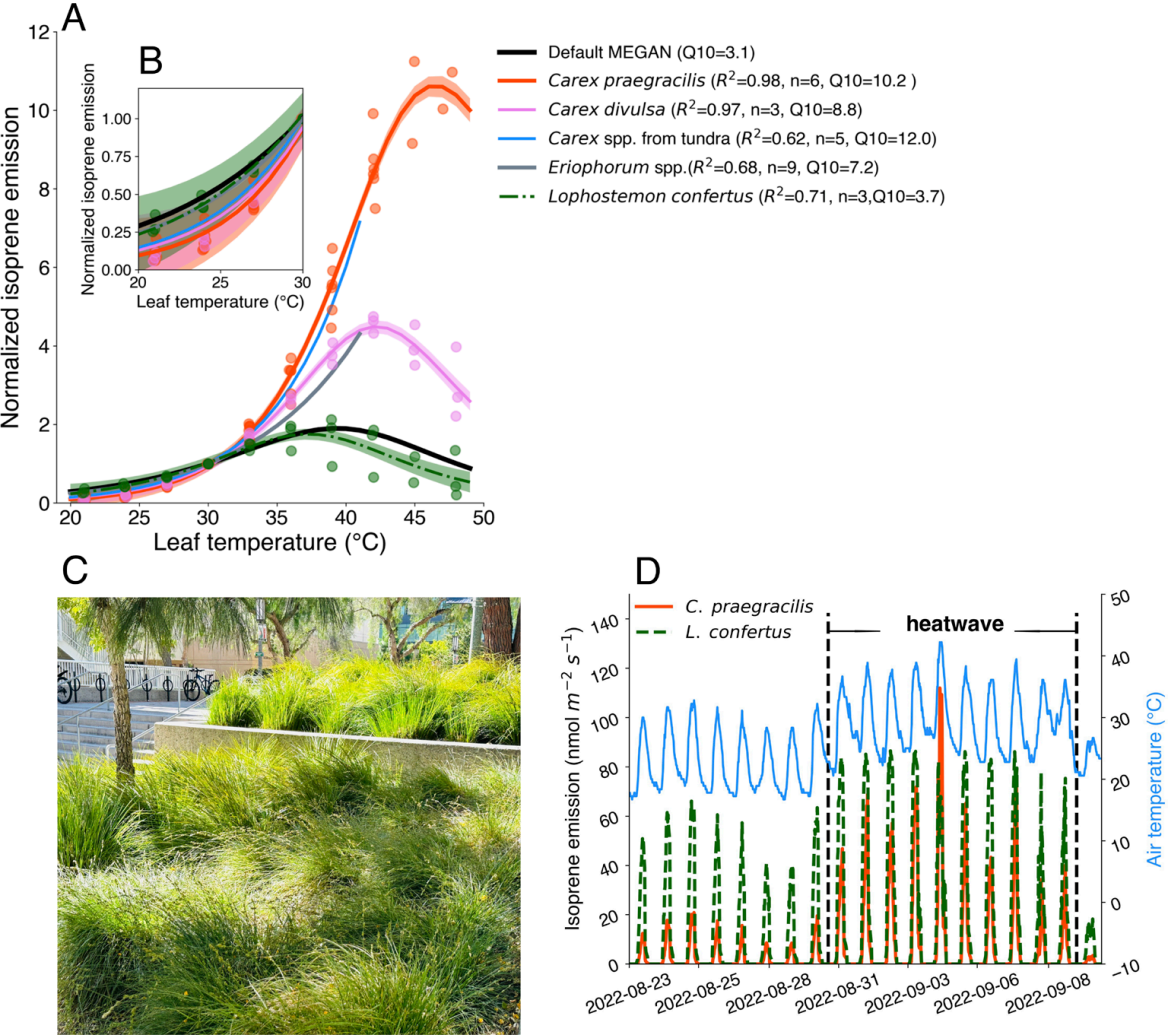
(*A* and *B*) Comparison of temperature response curves of sedges (*Carex* spp. and *Eriophorum* spp.), a typical tree (*L. confertus*), and the Model of Emissions of Gases and Aerosols from Nature version 2.1 (MEGANv2.1). The temperature response curves of *C. praegracilis* (orange solid line), *C. divulsa* (pink solid line), and *L. confertus* (green dashed line) were derived through leaf chamber experiments in this study. The circles and shadows represent the measurement points and 95% CI, respectively. The temperature response curves of *Carex* spp. (blue solid line) and *Eriophorum* spp. (gray solid line) from the Arctic are from Wang et al. (8). The temperature response curves are normalized to the emission level when the leaf temperature equals 30 °C (*SI Appendix*). The isoprene measurements below 35 °C, when isoprene emissions are more stable, are used to fit the Q10 values. (*C*) Urban landscapes covered by *C. praegracilis* (Photo credit: Hui Wang). (*D*) Comparison of isoprene fluxes estimated by MEGAN for the different temperature responses for *C. praegracilis* (orange solid line) and *L. confertus* (green dashed line) during the heat wave event in central Los Angeles during August to September 2022. The isoprene flux estimations are presented on the left axis, and the air temperature (blue solid line in *D*) changes are presented on the right axis.

We found that sedges from distinct climatic zones share a similar pronounced temperature response. Isoprene emissions from two sedge species that are widely distributed in temperate climate zones, *Carex praegracilis* and *Carex divulsa*, exhibited the same high sensitivity to temperature as previously observed for Arctic *Carex* spp. and *Eriophorum* spp. from northern Alaska (Fig. 1 *A* and *B*). All sedges showed a pronounced temperature response with a Q10 ranging from 7.2 to 12.0, which is much higher than that of other known isoprene emitters. For comparison, we used the same protocol and measured the temperature response curve of *Lophostemon confertus,* an evergreen broadleaf tree species that is widely planted in urban areas. The temperature response of *L. confertus* has a Q10 of 3.7, which is close to the Q10 of 3.1 in the widely used biogenic VOC emission model, the model of emissions of gases and aerosols from nature (MEGAN) (9), which proves that this high-temperature sensitivity is not caused by the protocol we used.

Further efforts were made to explore the cause of the high-temperature sensitivity of isoprene emissions from sedges in this study. When *C. praegracilis* and *C. divulsa* were exposed to a photosynthetic photon flux density (PPFD) level of 1,000 µmol m^−2^ s^−1^ for 8 h at a constant temperature of 21 °C (*SI Appendix*), the isoprene emissions from both species remained stable and did not increase. Therefore, this high-temperature sensitivity is not caused by extended exposure to a high light level. In addition, we observed that the isoprene emission from *C. praegracilis* went to nearly zero when the light was turned off at both low (21 °C) and high (42 °C) temperatures. This indicates that isoprene emission from sedges is light-dependent and is not from storage pool, suggesting that isoprene emissions from sedges are produced by the light-dependent synthesis of IspS. Wang et al. (8) observed that the pronounced temperature response curves of *Eriophorum* spp. in the Arctic could adapt to the growth temperature and eventually become similar to those of other normal isoprene emitters. They hypothesized that this high-temperature sensitivity behavior is related to the accumulation of IspS, in addition to the control of substrate supply and enzyme activities that are thought to control emissions of other isoprene emitters. However, the sedge species measured in this study, kept in a warm lab environment with a constant temperature of about 24 °C, still shows a pronounced temperature response curve. Field experiments, under a range of real-world conditions, and IspS activity assays, or measurements of postillumination isoprene release, could further illuminate the physiological processes of sedges.

**Fig. 2.**
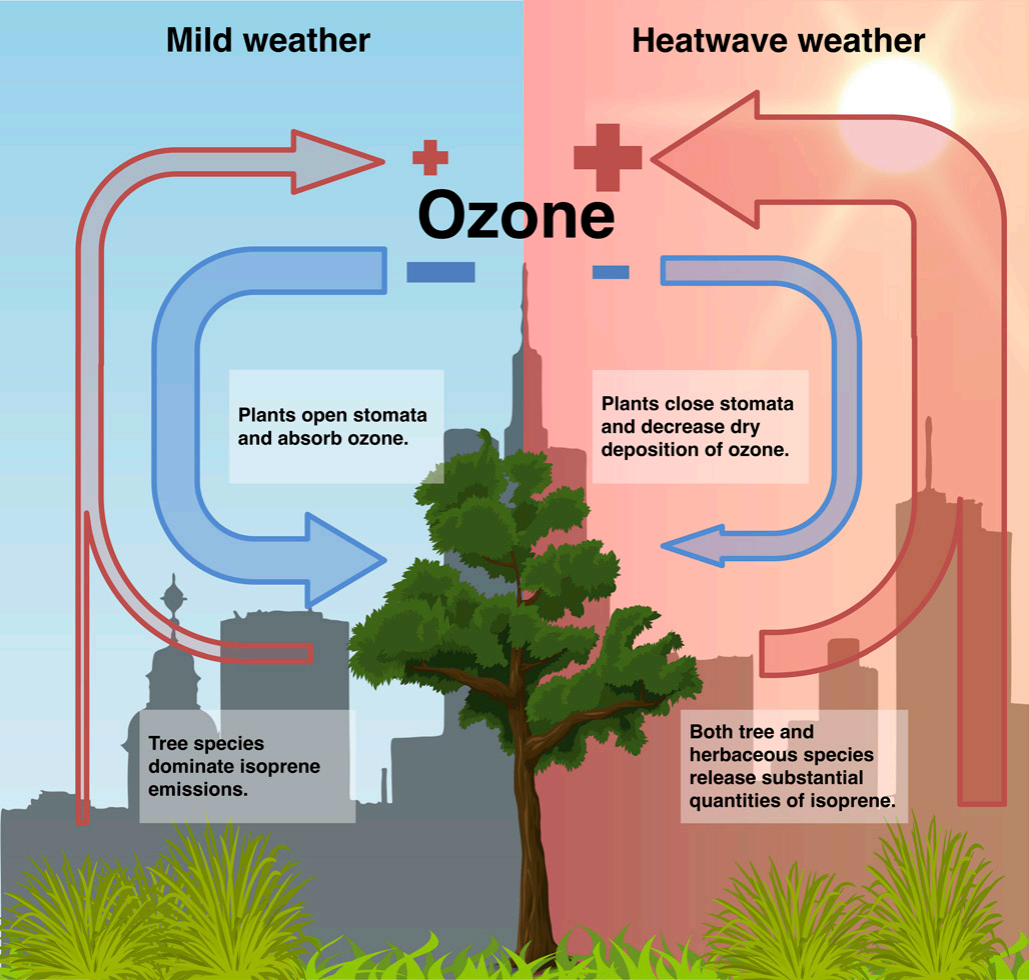
Conceptual figure illustrating the difference in the impact of vegetation on ozone pollution during mild and heat wave weather conditions. Both tree and herbaceous plants can release isoprene, a precursor to tropospheric ozone. During mild weather, isoprene emissions are primarily dominated by tree species, while herbaceous isoprene emitters have low emissions. However, during heat waves, isoprene emissions from both tree and herbaceous species significantly increase and can contribute about equally to the total emission. Additionally, plants tend to close their stomata during heat waves, reducing the dry deposition of ozone. The altered behavior of plants during heat waves can exacerbate ozone pollution in urban areas.

This study also indicates that graminoids could be a significant source of isoprene. With their high sensitivity to temperature changes, sedges could release large amounts of isoprene during heat waves when the environmental temperature is abnormally higher than the average. One species measured in this study, *C. praegracilis* (known as California Field Sedge or Clustered Field Sedge), is a native *Carex* species in North America and is widely used as a lawn substitute (Fig. 1*C*) (10). As an example of their potential contribution, we estimated the isoprene emissions from *C. praegracilis* and *L. confertus* during the heat wave that occurred from August to September 2022 in southern California. As shown in Fig. 1*D*, the average isoprene emission from *C. praegracilis* was 2.5 nmol m^−2^ s^−1^ before the heat wave with a maximum value of 21.6 nmol m^−2^ s^−1^. However, during the heat wave, the average isoprene emissions from *C. praegracilis* increased by 320% to 10.5 nmol m^−2^ s^−1^, which could account for about 56% of the isoprene emitted from *L. confertus* during the same heat wave event. The peak value of isoprene emissions from *C. praegracilis* could be 29% higher those from *L. confertus* with an emission rate of over 100 nmol m^−2^ s^−1^, even though the leaf area index (LAI) and the emission factor for *L. confertus* are higher than that for *C. praegracilis* (*SI Appendix*). This indicates that while isoprene emissions from sedges are low under mild weather conditions, they can become a significant source of isoprene during heat waves. Heat waves are often associated with peak air pollution events, especially ozone (5, 11). Previous studies demonstrated that current models failed to capture the ozone peak during heat waves in cities (12), where the chemistry regime is often VOC-limited and sensitive to changes in VOCs, especially isoprene. This study provides a potential explanation for high ozone concentrations during heat waves, which is not well-represented in current models. The high-temperature sensitivity of sedges makes them a hidden source of isoprene, not easily detected in mild weather but potentially significant during heat waves. Furthermore, heat waves can undermine the ability of plants to remove air pollutants, including ozone, by reducing stomatal conductance (13). Consequently, ecosystem and heat wave interactions could lead to an increase in ozone sources and a decrease in ozone sinks, as shown in Fig. 2, posing an extraordinary challenge to air pollution management with the increasing frequency of heat waves (14). As sedges are globally distributed and widely cultivated, these findings highlight the significance of isoprene emissions from herbaceous plants and their impact on atmospheric chemistry during heat waves and future warming.

## Materials and Methods

The temperature curve experiments for *C. praegracilis*, *C. divulsa*, and *L. confertus*. were conducted using a custom-made glass chamber with environmental control (15). The leaf-level isoprene emission was measured by proton transfer reaction-time of flight–mass spectrometry (PTR-TOF-MS) (1000 ultra; Ionicon Analytik, Austria). The experimental protocols are described in *SI Appendix*. Isoprene emission was estimated using the MEGAN model in Python with different temperature response curves, and the meteorological data used in this study were measured at central Los Angeles (34.10351 N, −118.26970 W) and are available through the MesoWest Database (https://mesowest.utah.edu/). Details about the experiments and model can be found in *SI Appendix*.

## Supplementary Material

Appendix 01 (PDF)

## Data Availability

Data and Python scripts for generating the figures in this study are archived and accessible through Zenodo (https://doi.org/10.5281/zenodo.12683973) (16). All other data are included in the manuscript and/or *SI Appendix*.
