# Supplementary material for: Heat waves may trigger unexpected surge in aerosol and ozone precursor emissions from sedges in urban landscapes
Source: Proc Natl Acad Sci U S A. 2024 Oct 21;121(45):e2412817121. doi: 10.1073/pnas.2412817121 (PMC11551377; doi:10.1073/pnas.2412817121)
Supplement: Supplementary file 1 — Appendix 01 (PDF) [file pnas.2412817121.sapp.pdf]

PNAS

Supporting Information for

Heatwaves may trigger unexpected surge in aerosol and ozone precursor emissions from sedges in urban landscapes

Hui Wang[1]*, Sanjeevi Nagalingam[1], Allison M. Welch[1], Christopher Leong[1], Claudia I. Czimczik[1], Alex B. Guenther[1]*

1.Department of Earth System Science, University of California, Irvine, California, USA

* Corresponding authors: Hui Wang and Alex B. Guenther

Email: huiw16@uci.edu and alex.guenther@uci.edu

This PDF file includes:

Supporting text

Temperature curve experiments

Vegetation samples. Three plant species were investigated. *Carex praegracilis* W. Boott (n=6) and *C. divulsa* Stokes (n=3) samples were potted plants, and the *Lophostemon confertus* (R. Br.) Peter G. Wilson & J.T. Waterh. samples (n=3) were branches cut from mature trees. The potted sedges were watered every day, including during the period when the experiments were conducted. *L.* confertus branch samples were collected from mature trees on the University of California Irvine campus. The branch stems were cut from three different trees at a height of 2 to 3 meters. They were all open canopy trees with some self-shading. The branch stems were cut, and immediately submerged under water in glass beakers and then recut, on the same day the experiments were conducted. We chose plants that were in good condition without galls or visible damage, and the leaves were taken from the second node and were about one year old.

Glass Leaf Chamber. The leaf-level isoprene measurements were conducted using a custom-made glass chamber with temperature and light controls. The glass chamber has an internal volume of 0.62 L, and a thermoelectric cooler assembly (Custom Thermoelectric, MD, USA) mounted to the chamber for precise control of leaf temperature. A miniature fan was installed at the bottom of the chamber to stir the air inside. A white LED source provided artificial illumination with a photosynthetic photon flux density (PPFD) of approximately 1000 µmol m^{-2} s^{-1}. Ambient air was pushed into the chamber using a diaphragm pump at rates of 0.9–1.0 L min^{-1}, measured with a flow meter, and the VOCs in the inlet airflow were removed by an activated carbon filter. Isoprene flux from the leaf inside the chamber was measured from the effluent air by Proton Transfer Reaction-Time of Flight–Mass Spectrometry (PTR-TOF-MS) (1000 ultra; Ionicon Analytik, Austria). Additionally, an infrared gas analyzer (LI-850; LI-COR Biosciences, NE, USA) was used to measure the CO_2 and H_2O mixing ratios in the influent (background) and effluent airflows; the analyzer was switched between the chamber's inlet and outlet every 30 seconds.

Temperature curve experiment protocol. The temperature curve experiments had three stages: control, darkness, and experiment. The control stage lasted 8 hours with a constant temperature of 21°C and a PPFD of 1000 µmol m^{-2} s^{-1}. Then, the light in the chamber is turned off for 8 hours to simulate a night environment, maintaining the same constant temperature of 21°C. After this darkness stage, the light is turned back on to 1000 µmol m^{-2} s^{-1}. The leaf temperature is kept at 21°C after the light is turned on and then ramped up to 42-51°C at a rate of 3°C hr^{-1}.

For *C. praegracilis* samples 1-4, we turned off the light when the leaf temperature reached 42°C to test if the isoprene is still light-dependent when the leaf temperature is high. For *C. praegracilis* samples 5-6, *C. divulsa* samples 1-3, and *L. confertus* samples.1-3, we heated up 48-51°C to observe the behavior of plants at extremely high temperatures. The PTR-TOF-MS continuously measured the isoprene concentrations from the chambers, and the average concentrations from the last 10 minutes at each temperature step were used to fit the temperature curves.

The Q10 value were fitted using Eq. [1]:

$$F = F_{30} \cdot Q10^{(T-30°C)/10} \quad [1]$$

, where F is the isoprene flux (nmol m^{-2} s^{-1}), F_{30} is the isoprene flux (nmol m^{-2} s^{-1}) when the leaf temperature equals 30°C, and T (°C) is the leaf temperature. The data below 35°C are used to fit the Q10 values for the increasing stage of isoprene emissions.

Isoprene emission modeling

We conducted two numerical simulation cases to understand the impact of different temperature curves on isoprene emission during heatwaves for C. *praegracilis* and *L. confertus*. As *C. praegracilis* is a widely distributed native species in North America that is used in the urban landscape of Southern California, we selected a heatwave event that occurred in Los Angeles, CA, USA, during August-September 2022.

MEGAN3-py model. The Model of Emissions of Gases and Aerosols from Nature version 3 in python (MEGANv3-py) was used in this study to estimate the isoprene emission from *C. praegracilis* and *L.*

confertus, respectively. MEGAN (1, 2) is a widely used biogenic volatile organic compounds (BVOCs) emission model, and the isoprene emission in MEGANv3 is estimated as [Eq. 2]:

$$F = \varepsilon \cdot LAI \cdot \gamma_T \cdot \gamma_P \cdot \gamma_A \cdot \gamma_C \cdot \gamma_D \quad [2]$$

, where ε, LAI, γ_T, γ_P, γ_A, γ_C, and γ_D represent the leaf-level standard emission factor (nmol m^{-2} s^{-1}), leaf area index (LAI, m^2 m^{-2}), and the environmental factors for the impact of temperature, light, leafage, CO_2, and drought. We focused on the temperature response of isoprene emission in this study, and the details about other factors can be found in previous studies (1-3).

The default short-term temperature response curve, γ_T, for isoprene in MEGANv3 is calculated as [Eq. 3]:

$$\gamma_{\mathrm{T}} = E_{\mathrm{opt}} \cdot \frac{C_{T2} \cdot e^{\frac{C_{T1}}{R}\left(\frac{1}{T_{\mathrm{opt}}} - \frac{1}{T}\right)}}{C_{T2} - C_{T1} \cdot \left(1 - e^{\frac{C_{T2}}{R}\left(\frac{1}{T_{\mathrm{opt}}} - \frac{1}{T}\right)}\right)} \quad [3]$$

, where *T (K)* is the leaf temperature (we used the air temperature as a proxy), R (=0.008314 kJ/mol) is the gas constant, and C_{T1} and C_{T2} are the activation and deactivation energies, respectively. The temperature curve parameters driving the *L. confertus* and *C. praegracilis* simulations were based on our measurements. When we fitted the temperature curves for each species, we normalized our measurements by dividing the isoprene emission at each temperature by the isoprene flux measured at 30°C for the same plant. This process reduces the impact of variability in isoprene emission capacity among different plants. The values of E_{opt}, C_{T1}, C_{T2}, and T_{opt} for *C. praegracilis* are 10.6 (± 0.5), 170.0 (± 33.2) kJ/mol, 260.7 (± 61.0) kJ/mol, and 319.6 (± 1.4) K, respectively. The values of E_{opt}, C_{T1}, C_{T2}, and T_{opt} for *C. divulsa* are 4.5 (± 0.3), 148.0 (± 36.7) kJ/mol, 288.0 (± 40.8) kJ/mol, and 315.4 (± 0.7) K, respectively. The values of E_{opt}, C_{T1}, C_{T2}, and T_{opt} for *L. confertus* are 1.8 (± 0.3), 114.7 (± 70.7) kJ/mol, 252.8 (± 69.3) kJ/mol, and 310.4 (± 2.1) K, respectively. The emission factors (ε) from our measurements for *C. praegracilis, C. divulsa,* and *L. confertus* are 6.3 (± 2.1), 21.0 (± 7.2), and 20.0 (± 7.8) nmol m^{-2} s^{-1}, respectively. We assigned a representative LAI of 4 for *L. confertus* and an LAI of 2.5 for *C. praegracilis* for the simulations.

The meteorology data was obtained from the Utah MesoWest Weather Data from the University of Utah and the National Weather Service Forecast Office in Salt Lake City (https://mesowest.utah.edu/) for the station (Station number: D1271, 34.10351 N, -118.26970 W) in central Los Angeles.

SI References

1. A. B. Guenther *et al.*, The Model of Emissions of Gases and Aerosols from Nature version 2.1 (MEGAN2.1): an extended and updated framework for modeling biogenic emissions. *Geoscientific Model Development* **5**, 1471-1492 (2012).

2. A. Guenther *et al.*, Estimates of global terrestrial isoprene emissions using MEGAN (Model of Emissions of Gases and Aerosols from Nature). *Atmos. Chem. Phys* **6**, 3181-3210 (2006).

3. H. Wang *et al.*, Modeling Isoprene Emission Response to Drought and Heatwaves Within MEGAN Using Evapotranspiration Data and by Coupling With the Community Land Model. *Journal of Advances in Modeling Earth Systems* **14**, e2022MS003174 (2022).
